# Longitudinal Multimodal Imaging Assessment of Tissue Changes Following Poly‐L‐Lactic Acid Treatment for Midfacial Rejuvenation: An Exploratory Pilot Study

**DOI:** 10.1111/jocd.71178

**Published:** 2026-09-21

**Authors:** Reem AL‐Attab, Jiajie Li, Chihchieh Lo, Hang Wang, Shuo Liu

**Affiliations:** ^1^ State Key Laboratory of Oral Diseases & National Center for Stomatology & National Clinical Research Center for Oral Diseases, Department of Maxillofacial Plastic, Aesthetic and Trauma Surgery West China Hospital of Stomatology, Sichuan University Chengdu Sichuan China

**Keywords:** collagen biostimulation, facial aging, midfacial rejuvenation, poly‐L‐lactic acid, three‐dimensional imaging, volume restoration

## Abstract

**Background:**

Poly‐L‐lactic acid (PLLA) is increasingly used as a collagen‐stimulating injectable for facial rejuvenation, but longitudinal multimodal characterization of volumetric and tissue remodeling changes after treatment remains limited.

**Aims:**

To characterize longitudinal changes in midfacial volume, ultrasound‐derived dermal remodeling parameters, and skin hydration after individualized PLLA treatment, and to explore the potential associations between administered PLLA amount and observed volumetric changes.

**Methods:**

Six adults with mild‐to‐moderate midfacial volume deficiency were enrolled in this prospective exploratory pilot study. Twelve hemifaces were treated with individualized PLLA injections over up to three sessions. Outcomes were assessed at baseline and Days 30, 90, 180, and 360 using 3D stereophotogrammetry, high‐frequency ultrasound, Corneometer measurements, standardized photography, and safety evaluation.

**Results:**

Exploratory analysis suggested a positive association between administered PLLA amount and observed volumetric changes at certain follow‐up time points, with the strongest association observed at Day 90. Mean volumetric increase was greatest at Day 360, whereas ultrasound‐derived dermal remodeling parameters and skin hydration peaked at Day 90 and remained elevated relative to baseline through Day 360. No serious treatment‐related adverse events were observed.

**Conclusions:**

In this exploratory cohort, individualized PLLA treatment was associated with longitudinal changes in midfacial volume and sustained alterations in ultrasound‐derived parameters related to dermal remodeling over 12 months. The observed association between administered PLLA amount and volumetric changes warrants further investigation in controlled studies to better understand factors contributing to variability in treatment outcomes.

## 1 Introduction

Facial aging is a natural process marked by textural and structural changes in the skin and underlying tissues [1, 2]. Over time, collagen depletion, fat redistribution, bone resorption, and gravitational effects lead to volume loss [3]. These factors produce signs of facial aging, including wrinkles, dehydration, diminished skin quality, and even changes in soft‐tissue distribution. Such changes can negatively impact psychological well‐being and social interactions by altering self‐perception and how others perceive one's appearance [4, 5].

Minimally invasive injection therapy has become increasingly routine and widespread because of its minimal trauma, reliable outcomes, and rapid recovery. It has emerged as a cornerstone of facial volume restoration, offering customizable, natural‐looking results [6, 7]. A wide range of injectable materials has been developed, broadly classified into physical fillers and biostimulator agents based on their mechanisms of action. Among these, poly‐L‐lactic acid (PLLA), a biostimulator with excellent biocompatibility, is now widely used in clinical practice [8]. Previous studies have suggested that PLLA may be associated with fibroblast‐mediated extracellular matrix remodeling, which may contribute to gradual changes in soft tissue quality and contour [9].

Although PLLA has demonstrated favorable clinical outcomes, its delayed tissue remodeling characteristics require careful treatment planning to achieve balanced aesthetic outcomes [10]. Treatment outcomes may be influenced by multiple factors, including treatment parameters, injection technique, and baseline tissue characteristics, highlighting the need for further investigation into factors associated with treatment variability [11]. However, longitudinal data exploring associations between treatment parameters and long‐term tissue changes following PLLA administration remain limited.

Therefore, this exploratory pilot study aimed to characterize longitudinal changes in midfacial volume, ultrasound‐derived dermal remodeling parameters, and skin hydration following individualized PLLA treatment, while exploring potential associations between administered PLLA amount and observed volumetric changes. Multimodal assessments, including 3D imaging, ultrasound‐derived evaluation, and standardized photography, were used to provide preliminary observations that may contribute to future investigations of factors associated with individualized treatment outcomes in facial rejuvenation.

## 2 Materials and Methods

### 2.1 Study Design

A prospective exploratory pilot study was conducted between December 2023 and August 2025. The study protocol was approved by the relevant ethics committee and was conducted in accordance with the principles of the Declaration of Helsinki. Written informed consent was obtained from all participants prior to treatment. Written consent for the use of clinical photographs was also obtained.

### 2.2 Participants

Six healthy adults were enrolled in this exploratory pilot study. Data from 12 treated hemifaces were included for longitudinal assessment. The participants included five females and one male, aged 22–53 years, all presenting with mild‐to‐moderate midfacial volume deficiency based on clinical assessment. As an exploratory pilot study, no formal sample size calculation was performed. The exclusion criteria were as follows: (1) hypersensitivity or a history of severe allergic reactions to local anesthetic agents or product components; (2) prior tissue regeneration treatments within 6 months; (3) recent cosmetic procedures, facial modifications, or augmentation with permanent or semi‐permanent fillers, autologous fat grafting, thread lifts, or implants; (4) active infections or skin lesions; (5) a history of cancer or prior radiation therapy in the treatment area; (6) recent use of chemotherapy, immunosuppressants, corticosteroids, local steroids, or systemic retinoids; (7) recent dental or oral surgery; (8) impaired wound healing (e.g., keloid formation or bleeding disorders); (9) significant facial asymmetry or congenital/acquired facial deformities that could interfere with outcome assessment; (10) inability to comply with study procedures; (11) current smoking; and (12) pregnancy or lactation. Participants were required to avoid cosmetic or surgical procedures that could affect study outcomes, including midface neurotoxin injections, chemical peels, and energy‐based treatments, for at least 6 months prior to enrollment and throughout the study period.

### 2.3 Injection Protocol

**FIGURE 1:**
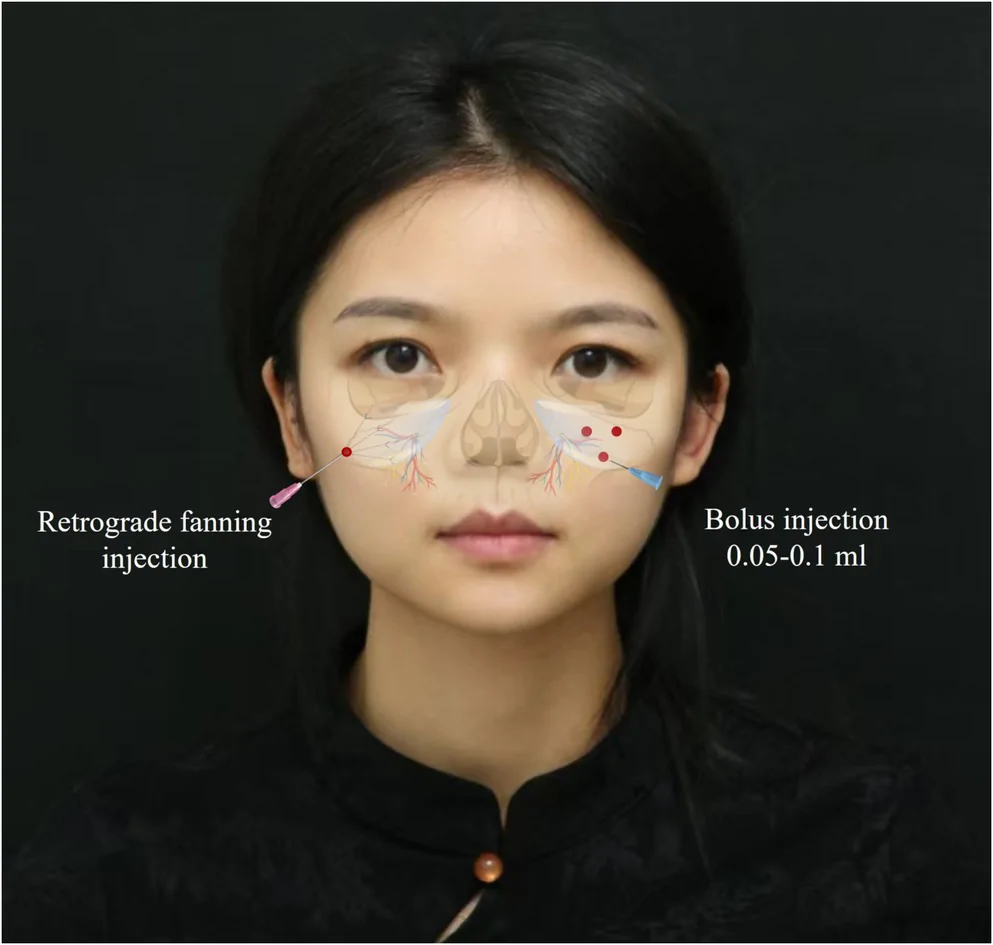
Schematic illustration of the injection technique used in the midface malar region, combining supraperiosteal needle bolus injections and cannula‐based retrograde fanning injections along the deep dermal–subcutaneous plane.

Each vial contained 170 mg of Löviselle (PLLA‐LaSynPro, Changchun SinoBiomaterials Co. Ltd., China), a PLLA filler composed of 75 mg PLLA microspheres, 22.5 mg sodium carboxymethyl cellulose (CMC), and 72.5 mg mannitol. For exploratory analysis, the total administered PLLA microsphere amount per hemiface was calculated as the cumulative mass delivered across all treatment sessions, ranging from 124 to 371 mg. Treatments were performed in the midface, comprising bolus injections targeting the medial and deep medial cheek fat compartments, and cannula‐based retrograde fanning injections distributing product across the lateral malar eminence, as illustrated in Figure 1. Needle bolus injections were placed in the supraperiosteal plane at predefined medial cheek points at three anatomical sites, whereas cannula‐based retrograde fanning injections were performed along the deep dermal–subcutaneous junction across the lateral malar region to achieve even product distribution. The administered amount at each session was individualized according to baseline midfacial volume deficiency and the treating physician's clinical assessment, reflecting routine clinical practice rather than a standardized dosing protocol.

### 2.4 Follow‐Up Visits

The study comprised eight scheduled visits (V0–V7). The baseline assessment visit (V0) included clinical and instrumental evaluations. This was followed by three injection visits (V1–V3) and four post‐treatment follow‐up visits (V4–V7). The first injection was administered at V1, followed by a second and third injection at intervals of 3–4 weeks. Follow‐up assessments, including clinical and instrumental evaluations, were conducted at 30 days (V4), 90 days (V5), 180 days (V6), and 360 days (V7) after the third injection. The third injection visit (V3) was administered to subjects whose clinical evaluation at V2 indicated residual volume deficit warranting further augmentation, as assessed independently by two investigators. Subjects achieving clinically acceptable midface volume restoration at V2 did not receive a third injection.

### 2.5 Quantitative Assessment

Measurements were performed in the bilateral malar regions of all participants at baseline (D0) and at 30 (D30), 90 (D90), 180 (D180), and 360 (D360) days after the third injection. To ensure measurement accuracy and reproducibility, three consecutive scans were obtained at each assessment site, and the mean value was used for analysis.

#### 2.5.1 3D Vectra H1

Three‐dimensional facial volumetric assessments were performed using the Vectra H1 imaging system (Canfield Scientific Inc., USA). Volumetric changes in the treated midface region were calculated at D30, D90, D180, and D360 relative to baseline. Mean volumetric changes were recorded at each follow‐up visit. Exploratory association analyses were performed to evaluate the potential association between administered PLLA amount and observed volumetric changes at each assessment time point.

#### 2.5.2 Ultrasound‐Derived Dermal Remodeling Parameter

The ultrasound‐derived dermal remodeling parameter was assessed using the DermaLab Aesthetic high‐resolution ultrasound system (Cortex Technology, Denmark). The parameter is derived from ultrasound signal intensity measurements and was used as a noninvasive imaging‐based surrogate marker of dermal remodeling. It does not directly quantify collagen content or collagen synthesis. Ultrasound measurements were performed in the treated malar region at baseline and each follow‐up visit. Changes in the ultrasound‐derived dermal remodeling parameter over time were recorded and analyzed to evaluate dermal remodeling following treatment.

#### 2.5.3 Skin Hydration

Skin hydration was assessed using the Corneometer CM 825 (Courage + Khazaka Electronic GmbH, Germany), a capacitance‐based instrument widely used for quantitative evaluation of skin hydration. Measurements were performed in the treated malar region at each assessment visit and recorded in Corneometer units (C.U.). Higher values indicate greater skin hydration.

### 2.6 Qualitative Assessment

#### 2.6.1 2D Photograph

Standardized facial photographs were obtained using an EOS 6D digital camera (Canon Inc., Japan) at baseline and each follow‐up visit. Photographs were acquired under standardized lighting, positioning, and camera settings to provide visual documentation of treatment‐related aesthetic changes over time.

#### 2.6.2 Participant Satisfaction Assessment

Participant‐reported satisfaction was collected during follow‐up visits as a descriptive subjective assessment of participants' perceptions following treatment. Participants were asked about their overall satisfaction with aesthetic changes and perceived changes in skin texture. Responses were recorded descriptively and summarized at each follow‐up visit.

### 2.7 Adverse Event Assessment

Adverse events occurring during and after treatment, including pain, bruising, erythema, skin irritation, pruritus, and burning sensation, were recorded throughout the study period. Event severity was categorized as mild (easily tolerated), moderate (causing noticeable discomfort), or severe (causing significant discomfort or requiring intervention). Adverse events were summarized descriptively at the participant level.

### 2.8 Statistical Analysis

Statistical analyses were performed using GraphPad Prism (Version 9.0; GraphPad Software, Boston, MA, USA). Hemiface‐level analyses were conducted using individual hemifaces as the primary anatomical unit (*n* = 12). Because bilateral hemifaces from the same participant are anatomically paired and not fully statistically independent, hemiface‐level analyses were considered exploratory. To further explore the pattern of the observed association between administered PLLA amount and volumetric changes, a subject‐level aggregated sensitivity analysis was also performed by combining bilateral injected PLLA doses and bilateral volumetric changes within each participant (*n* = 6). Participant‐reported outcomes and safety data were summarized descriptively at the participant level. Data distribution was assessed using the Shapiro–Wilk test. Changes in volumetric measurements, ultrasound‐derived dermal remodeling parameter, and skin hydration over time were evaluated using repeated‐measures ANOVA for normally distributed data and the Friedman test for non‐normally distributed data. Post hoc pairwise comparisons were performed when overall significance was observed. Because of the exploratory nature and limited sample size of this pilot study, descriptive statistics, temporal trends, and effect‐size estimates were emphasized over formal hypothesis testing. To explore potential associations between administered PLLA amount and observed volumetric changes, Pearson correlation analyses were performed at each assessment time point. Exploratory correlations involving ultrasound‐derived dermal remodeling parameter and skin hydration were evaluated descriptively when applicable. Given the exploratory nature and limited sample size of this pilot study, all correlation analyses were considered hypothesis‐generating and should not be interpreted as evidence of a causal or definitive dosing model. A two‐sided *p* value < 0.05 was considered statistically significant for descriptive comparisons.

## 3 Results

### 3.1 Longitudinal Volumetric Changes by Three‐Dimensional Imaging

**FIGURE 2:**
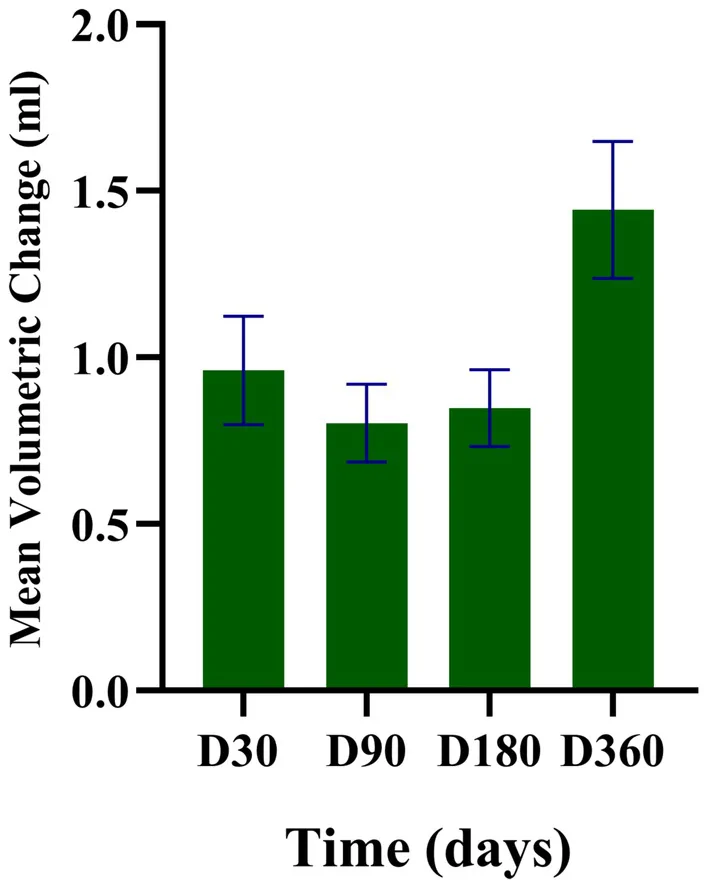
Mean volumetric changes relative to baseline at each follow‐up visit after PLLA treatment. Volumetric changes were assessed using the Vectra H1 three‐dimensional stereophotogrammetry system. Bars represent mean volumetric changes relative to baseline, and error bars indicate standard deviation.

**FIGURE 3:**
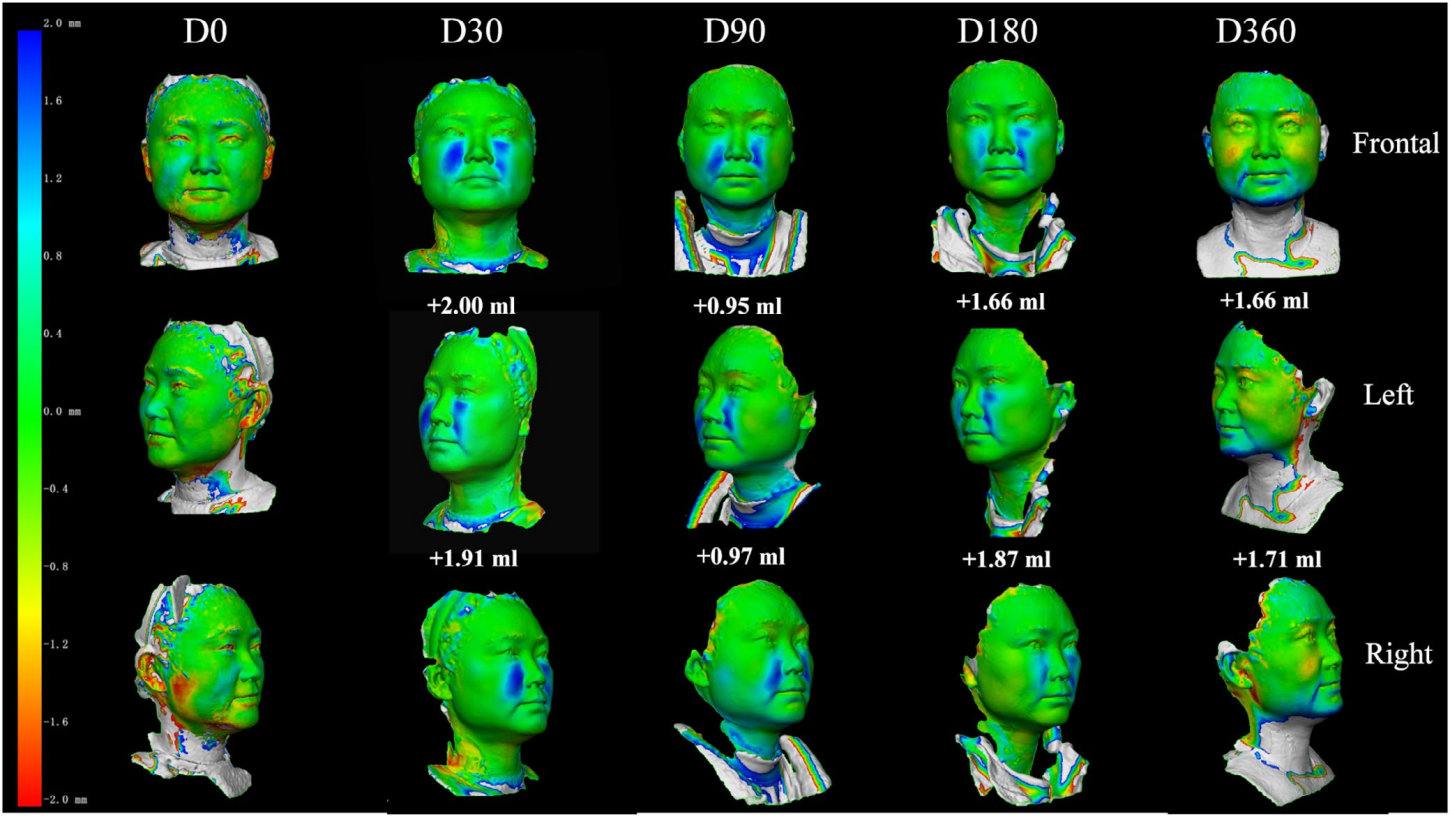
Representative three‐dimensional color distance maps acquired using the Vectra H1 stereophotogrammetry system. Images illustrate longitudinal surface displacement and surface differences in the treated midfacial region of a 44‐year‐old female who received a total of 205 mg PLLA over three treatment sessions performed at 3–4‐week intervals. Blue regions indicate positive surface displacement relative to baseline, whereas yellow regions represent additional surface differences detected by three‐dimensional surface analysis.

As shown in Figure 2, volumetric increases were observed at all follow‐up visits compared with baseline (D0). Mean volumetric changes were 0.96 mL, 0.80 mL, 0.85 mL, and 1.44 mL at D30, D90, D180, and D360, respectively. The largest mean volumetric increase was observed at D360. A Friedman test indicated differences in volumetric measurements across follow‐up visits (*p* < 0.05); however, given the exploratory design and limited sample size, these findings were interpreted primarily as descriptive temporal trends. Figure 3 presents a representative three‐dimensional color distance map obtained using the Vectra H1 3D photogrammetry system from a 44‐year‐old female who received a total of 205 mg PLLA over three treatment sessions in the midface region. The three‐dimensional color distance map demonstrated temporal changes in surface morphology relative to baseline (D0) throughout the follow‐up period. Positive surface displacement was represented by blue regions in areas corresponding to the treated midfacial region. At the final follow‐up visit, volumetric increases were similar on both sides of the face, measuring 1.71 mL on the right side and 1.66 mL on the left side. Additional surface contour differences were observed at D360, as indicated by the yellow regions surrounding the treatment area.

### 3.2 Ultrasound‐Derived Dermal Remodeling Parameter

**FIGURE 4:**
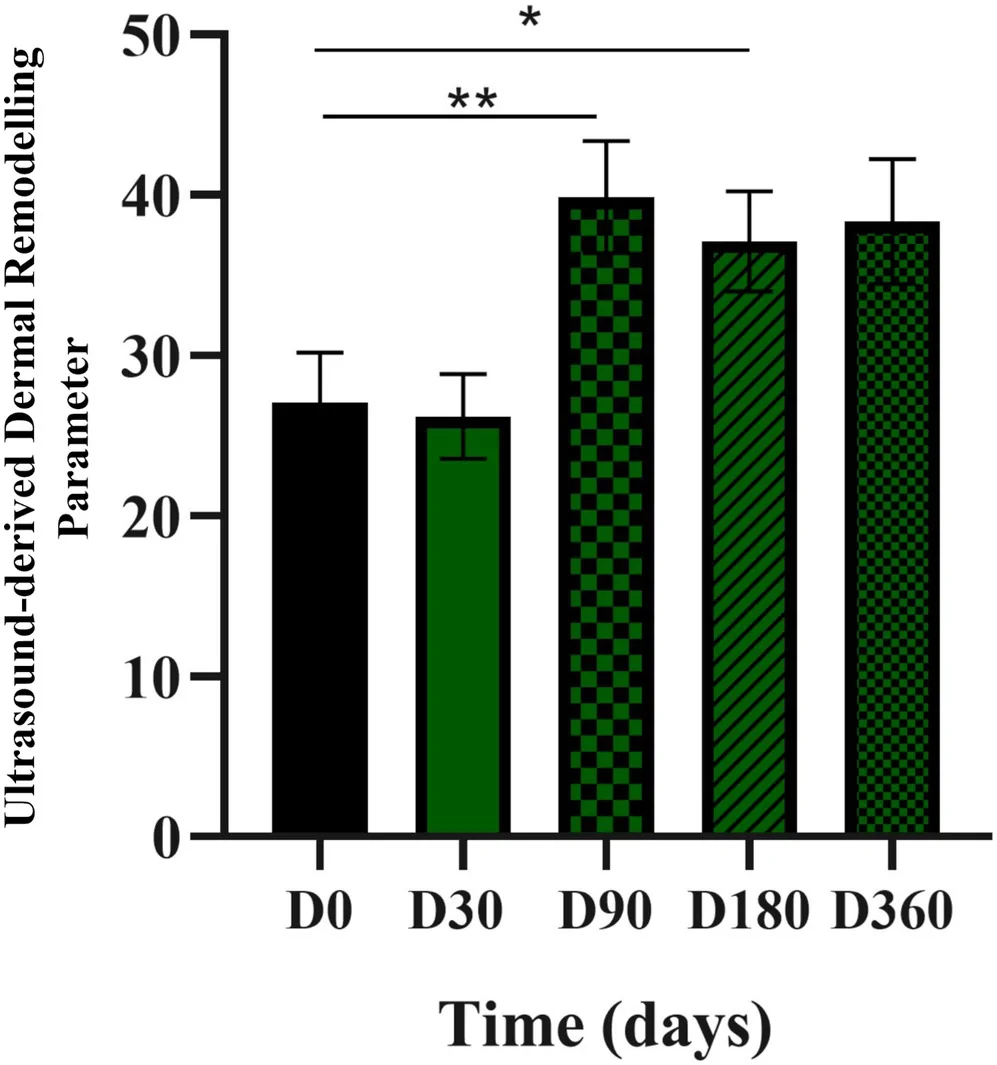
Ultrasound‐derived dermal remodeling parameter values measured by DermaLab high‐resolution ultrasound at each follow‐up visit. Bars represent mean parameter values, and error bars indicate standard deviation. Statistical significance is indicated by connecting lines and asterisks (*p* < 0.05).

**FIGURE 5:**
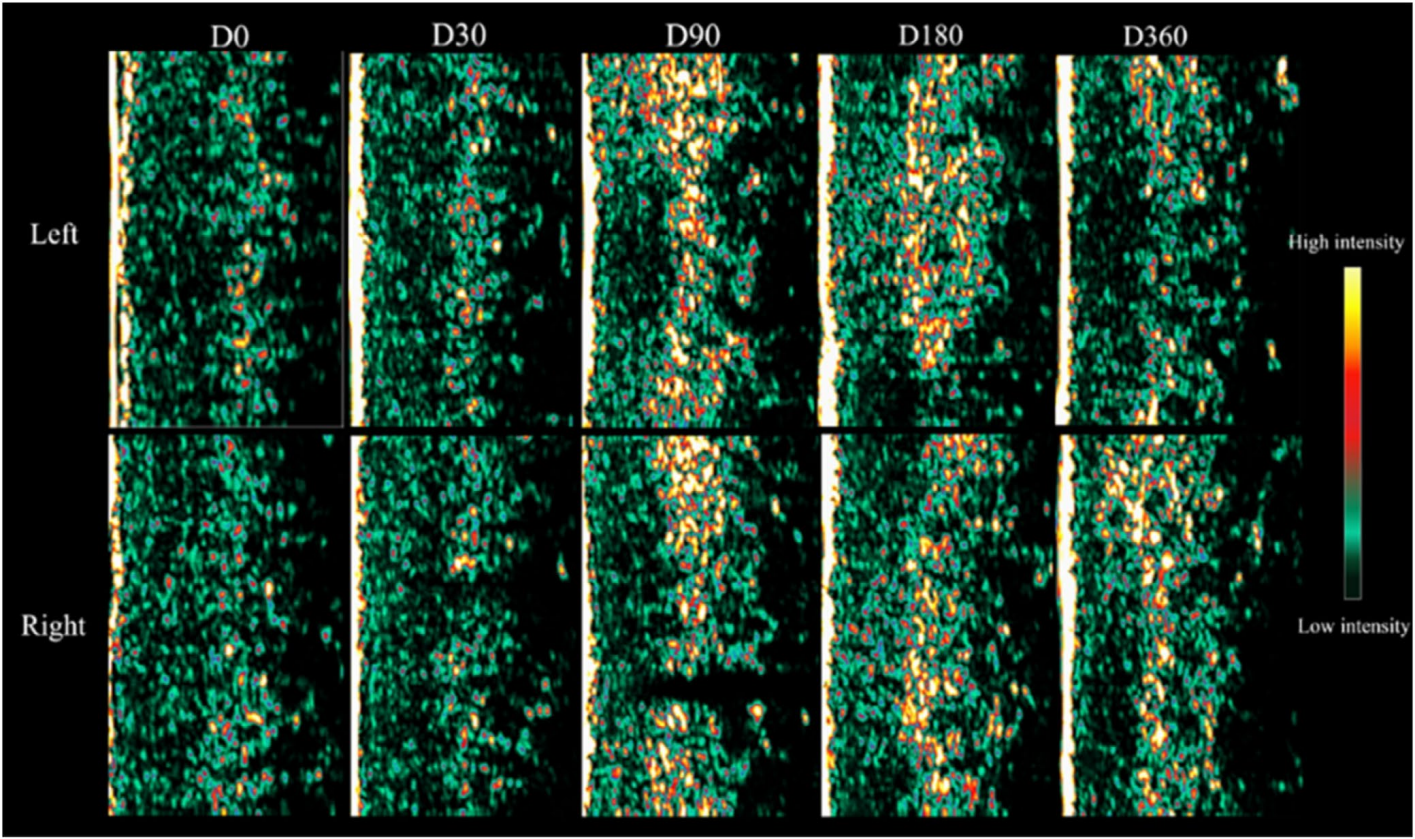
Representative DermaLab high‐resolution ultrasound cross‐sectional images obtained at different follow‐up time points after PLLA treatment. Images illustrate temporal changes in dermal signal intensity corresponding to the quantitative ultrasound‐derived parameter values presented in Figure 4. Red and yellow pixels indicate areas of relatively higher ultrasound signal intensity, whereas green pixels indicate areas of relatively lower signal intensity.

As shown in Figure 4, high‐resolution ultrasound measurements demonstrated temporal changes in an ultrasound‐derived dermal remodeling parameter based on signal intensity measurements during follow‐up. Compared with baseline (D0), no significant increase was observed at D30. Mean ultrasound‐derived dermal remodeling parameter reached 39.86 ± 11.65 at D90, 37.11 ± 10.39 at D180, and 38.33 ± 12.92 at D360, representing increases of approximately 47%, 37%, and 41%, respectively, relative to baseline. The highest mean ultrasound‐derived dermal remodeling parameter was observed at D90, with elevated values remaining at subsequent follow‐up visits. These findings were interpreted descriptively because the ultrasound‐derived parameter represents an indirect imaging marker rather than a direct measurement of collagen formation. In Figure 5, representative DermaLab high‐resolution ultrasound cross‐sectional images from a 44‐year‐old female demonstrate temporal changes in dermal signal intensity following PLLA treatment. The dermal layer is represented by green pixels, while areas of higher signal intensity are represented by red and yellow pixels. Increased signal intensity patterns were observed at D90 and remained relatively elevated at subsequent follow‐up visits, corresponding to the quantitative changes in the ultrasound‐derived parameter shown in Figure 4.

### 3.3 Skin Hydration

**FIGURE 6:**
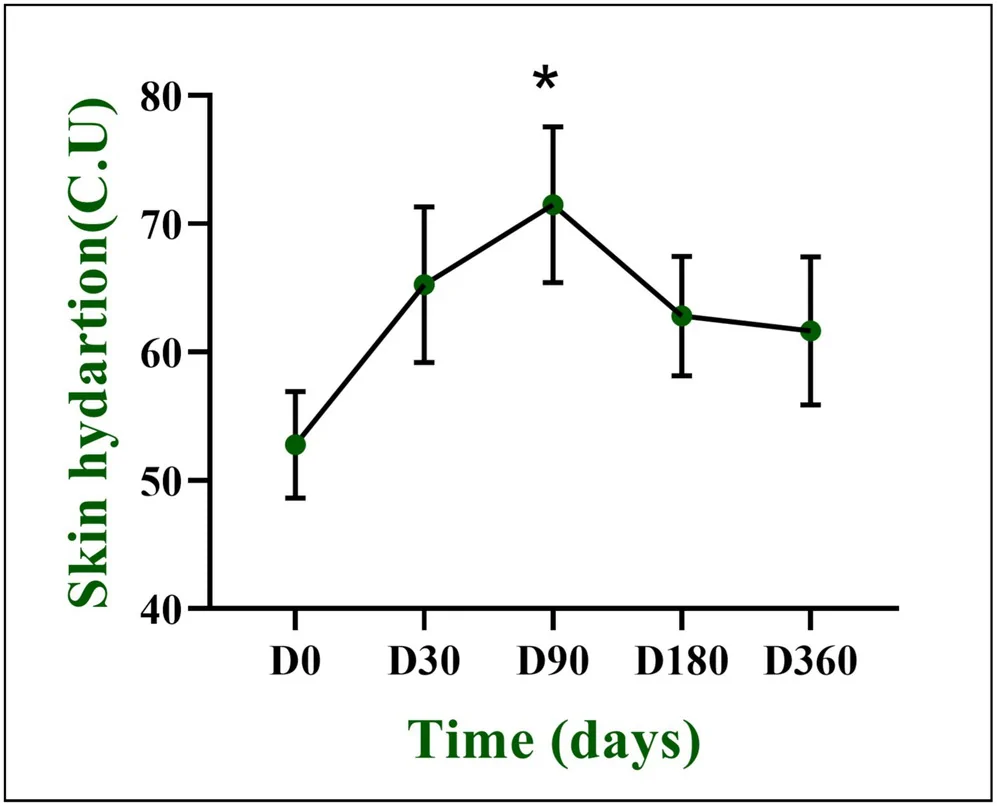
Mean skin hydration measured by Corneometer CM 825 at each follow‐up visit. Bars represent mean hydration values, and error bars indicate standard deviation. Statistical significance is indicated by connecting lines and asterisks (*p* < 0.05).

As shown in Figure 6, Corneometer measurements demonstrated temporal changes in skin hydration during follow‐up. Baseline skin hydration was 50.22 ± 12.38 C.U. Mean hydration values were 65.34 ± 20.55 C.U. at D30, 72.10 ± 19.63 C.U. at D90, 60.84 ± 13.45 C.U. at D180, and 65.28 ± 14.55 C.U. at D360, corresponding to increases of approximately 30%, 44%, 21%, and 30%, respectively, relative to baseline. The highest mean hydration value was observed at D90. Differences between D0 and D90, and between D0 and D180, reached nominal statistical significance (*p* < 0.05). Because of the exploratory design and limited sample size, these *p* values should be interpreted descriptively.

### 3.4 Exploratory Analysis of the Association Between Administered PLLA Amount and Volumetric Changes

**FIGURE 7:**
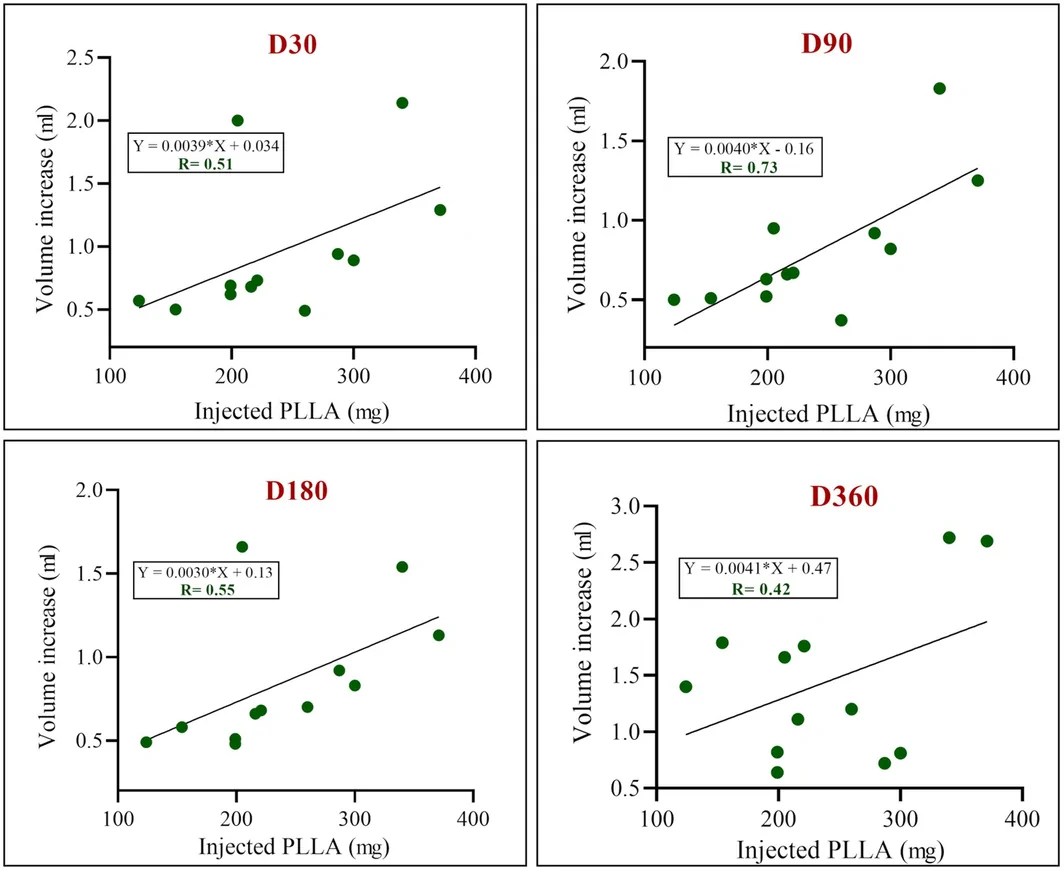
Exploratory associations between administered PLLA amount and observed volumetric changes at each follow‐up time point. Scatter plots show the association between administered PLLA amount and volumetric changes at D30, D90, D180, and D360. Corresponding exploratory linear regression lines, equations, and Pearson correlation coefficients (*r*) are presented for each time point.

Exploratory analyses suggested positive associations between the administered PLLA amount and observed volumetric changes across follow‐up time points (Figure 7). At D30, a moderate positive association was observed between cumulative administered PLLA amount and volumetric increase (*R* = 0.51). The strongest association was identified at D90 (*R* = 0.73). Moderate positive associations were also observed at D180 (*R* = 0.55) and D360 (*R* = 0.42). Overall, within this exploratory dataset, higher administered PLLA amounts were numerically associated with greater observed volumetric changes; however, this observation may be influenced by individualized treatment allocation and baseline volume deficiency. The strength of the association varied over time, with the highest correlation coefficient observed at D90 and lower correlations at D30, D180, and D360.

Similar temporal patterns were observed in the subject‐level aggregated sensitivity analysis, in which bilateral hemiface data were aggregated within each participant (Table S1). In this analysis, the strongest association was also observed at D90 (*R* = 0.71), whereas the association at D360 was weaker (*R* = 0.22), potentially reflecting greater variability at later follow‐up time points and the inherent instability of correlation estimates in very small samples. Given the exploratory nature of this pilot study, these observed dose–volume associations should be interpreted as hypothesis‐generating rather than evidence of a causal or definitive dose–response association.

### 3.5 Standardized Photography and Participant Satisfaction

**FIGURE 8:**
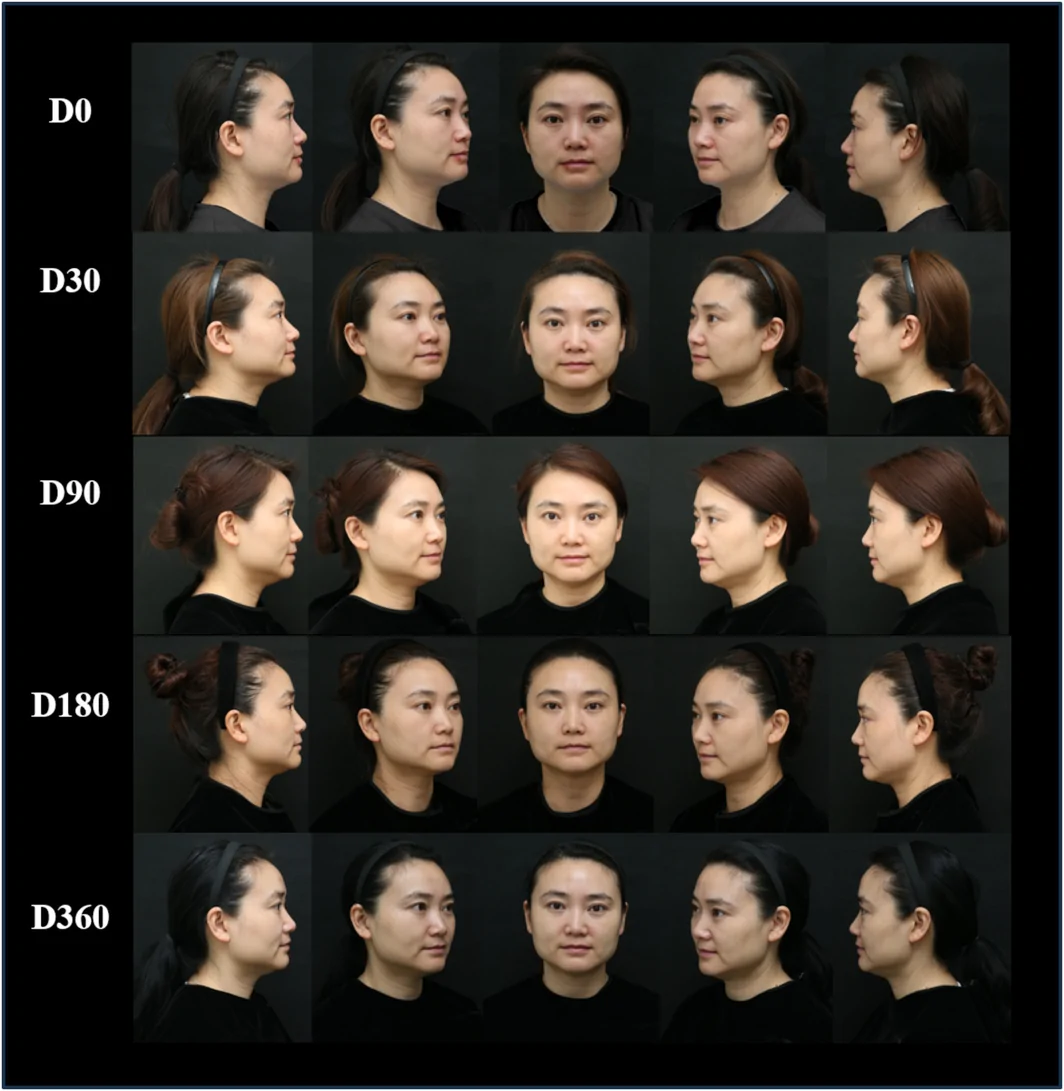
Representative longitudinal standardized two‐dimensional clinical photographs of a 44‐year‐old female following PLLA treatment. The participant received a total of 205 mg PLLA over three treatment sessions performed at 3–4‐week intervals. Photographs were obtained at baseline (D0) and follow‐up visits through D360.

Qualitative assessment of standardized clinical photographs demonstrated visible changes in midface contour and volume during follow‐up after PLLA treatment (Figure 8). In this representative case, visual changes in midface projection and soft tissue fullness were observed after treatment and remained apparent through D360. No obvious asymmetry or excessive volumization was observed in this representative case during follow‐up.

Participant feedback collected during follow‐up visits indicated generally favorable perceptions of treatment outcomes. Participants generally reported favorable perceptions of overall aesthetic improvement and improvements in skin texture following treatment. One participant reported no noticeable improvement in skin texture. Overall, participant‐reported outcomes provided complementary subjective assessments of perceived aesthetic changes during follow‐up.

### 3.6 Safety

No unexpected or serious treatment‐related adverse events were observed during the study. Mild injection‐site reactions, including transient redness, pain, and swelling, were observed in several participants and resolved spontaneously without intervention. One participant experienced moderate bruising accompanied by pain and swelling, which resolved without sequelae. No nodules, granulomas, vascular complications, or other severe adverse events were observed during the follow‐up period.

## 4 Discussion

Facial volume gradually decreases with age owing to underlying bone and fat resorption, which is exacerbated by reduced collagen content and synthesis [1, 2]. These changes degrade skin texture and overall dermal quality, contributing to lower self‐esteem. This influences how individuals are socially evaluated and affects mental and social well‐being [12]. In response to these age‐related concerns, the demand for aesthetic procedures has risen significantly in recent decades [13, 14]. Because skin aging is multifactorial and has multiple underlying causes, different products and methods need to address the various contributing factors effectively [15].

Although the benefits of PLLA fillers are well documented [16], longitudinal patterns of tissue changes following individualized PLLA treatment and factors associated with variability in treatment outcomes remain incompletely characterized. In this study, we characterized longitudinal changes in midfacial volume, ultrasound‐derived dermal remodeling parameters, and skin hydration following individualized PLLA treatment using multimodal assessment tools, including 3D Vectra imaging, DermaLab high‐resolution ultrasound, and standardized photography, while exploring potential associations between administered PLLA amount and observed volumetric changes. These findings provide preliminary observations regarding temporal patterns of tissue response and may inform future studies investigating factors associated with individualized PLLA treatment outcomes.

Exploratory analysis using 3D Vectra H1 identified the strongest observed association between administered PLLA amount and volumetric changes at D90 (*R* = 0.73), whereas moderate positive associations persisted at D180 and D360. These findings suggest that this association between administered PLLA amount and volumetric changes varied across follow‐up time points; however, the underlying factors contributing to this pattern remain uncertain. Because cumulative dose was determined based on baseline midfacial volume deficiency and clinical treatment needs rather than randomly assigned, the observed dose–volume associations may partly reflect baseline severity and treatment allocation decisions rather than a pure pharmacologic dose–response association. Given the exploratory design and individualized dosing strategy used in this study, these observations should be interpreted as hypothesis‐generating rather than evidence of a definitive dose–response model. Accordingly, the statistical analyses were intended to characterize temporal trends and the magnitude of exploratory associations rather than to provide confirmatory inference. Beyond volumetric outcomes, previous literature has suggested that the long‐term tissue response following PLLA treatment may involve extracellular matrix remodeling processes, including collagen‐related changes and matrix reorganization [17, 18]. In addition, the persistence of volumetric changes at D360 may indicate that tissue responses continued beyond the early post‐treatment period, although the specific biological mechanisms underlying these changes require further investigation. Therefore, individualized treatment approaches remain an important area for future research, although larger controlled studies are required to determine how treatment parameters influence outcomes.

The greatest mean volumetric increase was observed at D360 (approximately 1.5 mL) [19]. The initial volume increase at D30 may be partly attributable to early post‐injection tissue response and transient edema, as previously reported [20]. The slight decrease in volume at D90 may reflect temporal variability in tissue response following treatment [21]. Notably, the highest ultrasound‐derived dermal remodeling parameter was observed at D90, whereas the greatest volumetric increase occurred at D360. This temporal dissociation suggests that imaging‐derived remodeling‐related changes and volumetric changes may follow different temporal patterns after PLLA treatment. The late‐stage volume increase observed at D360 may reflect continued tissue adaptation and remodeling‐related changes after treatment [22]. The representative 3D Vectra color‐distance maps were consistent with the quantitative volumetric findings. The blue regions in the treated midfacial region indicated positive surface displacement relative to baseline over time. Additional topographic differences observed at D360 may reflect continued changes in tissue contour over time. The sustained volumetric changes observed through 1 year suggest that the measured changes may involve longer‐term tissue responses beyond the early post‐injection period, although the specific mechanisms require further investigation [23].

The ultrasound‐derived dermal remodeling parameter showed temporal increases during follow‐up, particularly at D90, and remained elevated through D180 and D360. This observation is consistent with previous reports describing PLLA‐associated extracellular matrix remodeling [24]. Bravo et al. reported that PLLA injections may trigger a controlled foreign‐body response associated with progressive collagen remodeling, with peak biological activity occurring approximately 3 months after treatment [25]. Furthermore, DermaLab skin cross‐sectional images were generally consistent with the quantitative measurements obtained in this study. Because this ultrasound‐derived parameter is based on signal intensity measurements, it should be interpreted as a noninvasive surrogate marker of dermal remodeling rather than direct histological evidence of collagen deposition or neocollagenesis [26]. An increase in dermal red and yellow pixel intensity was observed at D90, coinciding with the strongest dose–volume association. This temporal pattern suggests that imaging‐derived remodeling‐related changes and volumetric changes may evolve along partially distinct trajectories after PLLA treatment. Although not directly demonstrated by the ultrasound measurements in the present study, previous studies have suggested that PLLA may promote extracellular matrix remodeling through fibroblast‐mediated signaling pathways, including PI3K/AKT activation [27]. Moreover, Christen et al. discussed macrophage‐associated remodeling processes as potential mechanisms contributing to PLLA‐related dermal matrix changes and improvements in skin quality [18].

Skin hydration measurement showed a gradual improvement, peaking at D90. Interestingly, the maximal hydration values occurred during the same period in which the ultrasound‐derived dermal remodeling parameter reached its highest level, which may suggest a temporal association between dermal remodeling and skin hydration status. However, PLLA itself does not possess intrinsic water‐binding properties comparable to hyaluronic acid and therefore should not be regarded as a direct moisturizing agent. Instead, changes in dermal properties, extracellular matrix organization, and skin barrier‐related factors may contribute to increased hydration measurements detected by the Corneometer. A recent comprehensive literature review confirmed the efficacy of PLLA in improving skin hydration, with noticeable enhancement beginning at approximately 3 months and persisting for up to 12 months [16]. Although hydration levels slightly decreased at D180, this fluctuation may be influenced by endogenous and exogenous factors, including physiological variation, seasonal changes, and alterations in skin barrier function over time, as described by Munia et al. [28].

Visual changes in midfacial contour and soft tissue appearance were observed in standardized 2D photographs during follow‐up. Participants treated within the studied dose range demonstrated visually apparent volumetric changes without obvious signs of overcorrection in the available clinical assessments, supporting the feasibility of individualized treatment approaches in clinical practice [29]. Moreover, participants reported favorable perceptions of treatment outcomes, particularly regarding the skin appearance and facial contour improvement. Adverse events were predominantly mild and transient, further supporting the safety profile of this PLLA formulation in the studied population, as previously reported [30].

Clinically, our findings highlight the value of longitudinal multimodal assessment for characterizing the evolving effects of PLLA biostimulator treatment. The observed temporal patterns suggest that different outcome measures, including volumetric changes and ultrasound‐derived remodeling‐related parameters, may exhibit distinct trajectories following treatment, and that treatment effects may become more apparent over extended follow‐up periods. Although these findings should not be interpreted as establishing a definitive dosing algorithm, they provide preliminary observations that may inform future prospective studies investigating individualized treatment approaches and factors associated with clinical outcomes.

This study has several limitations despite the preliminary findings. First, the study included only six participants and was designed as an exploratory pilot investigation; therefore, the findings require confirmation in larger cohorts. Second, the use of individualized dosing reflects real‐world clinical practice but may introduce confounding when evaluating dose–response associations. Specifically, because higher cumulative doses were generally administered to participants with greater baseline midfacial volume deficiency or greater clinically assessed treatment needs, baseline severity and treatment allocation decisions may have contributed to the observed dose–volume associations. Therefore, these findings should not be interpreted as establishing a causal or pharmacologic dose–response association. Third, the absence of a control group limits causal interpretation of the observed longitudinal changes and prevents differentiation between PLLA‐associated effects and potential influences from natural temporal variation, measurement variability, or other time‐dependent factors. Fourth, the ultrasound‐derived dermal remodeling parameter was assessed using an ultrasound‐derived imaging parameter rather than direct histological evaluation. Finally, although the formulation contains both PLLA and sodium carboxymethyl cellulose (CMC), the relative contribution of each component to early volumetric changes cannot be completely distinguished in the present study. Nevertheless, the sustained volumetric changes observed through D360, together with the temporal pattern of ultrasound‐derived dermal remodeling parameters, suggest that the observed long‐term changes cannot be fully attributed to the initial physical contribution of the CMC‐containing formulation alone. Future studies with larger sample sizes, randomized populations, multiple treatment areas, and histological validation are needed to better characterize factors influencing treatment outcomes and clarify the biological mechanisms underlying observed tissue changes.

## 5 Conclusion

This exploratory pilot study characterized longitudinal changes in midfacial volume, ultrasound‐derived dermal remodeling parameters, and skin hydration following individualized PLLA treatment, while exploring potential associations between administered PLLA amount and observed volumetric changes. The strongest association between administered PLLA amount and volumetric changes was identified at Day 90, coinciding with the highest mean value of the ultrasound‐derived dermal remodeling parameter. These temporal patterns suggest that different outcome measures may exhibit distinct trajectories following PLLA treatment; however, the observed associations should be interpreted as exploratory and hypothesis‐generating.

Within the limitations of this exploratory study, future studies are needed to identify factors associated with variability in clinical outcomes following PLLA‐based biostimulation. These findings provide preliminary observations that may inform future controlled studies investigating factors associated with treatment outcomes and highlight the value of longitudinal multimodal assessment for characterizing the evolving effects of biostimulatory fillers.

## Author Contributions

Reem AL‐Attab designed the study, performed the research, analyzed data, and wrote the paper. Hang Wang designed and performed the research; Shuo Liu designed and performed the research; Jiajie Li contributed to refining the ideas; Chihchieh Lo conducted additional analyses.

## Funding

The authors have nothing to report.

## Disclosure

This manuscript has not been previously published and is not under consideration for publication elsewhere.

## Ethics Statement

All patients provided written, informed consent. All procedures were approved by the Institutional Review Board (Approval No. WCHSIRB‐D‐2023‐219). Procedures operated in this research were completed in keeping with the standards set out in the Declaration of Helsinki and laboratory guidelines of research in China.

## Conflicts of Interest

The authors declare no conflicts of interest.

## Supporting information


**Table S1:** Exploratory analyses of the relationship between administered PLLA amount and volumetric changes at different follow‐up time points.

## Data Availability

The data supporting the findings of this study can be obtained from the corresponding author upon reasonable request.
