# Supplementary material for: Longitudinal Multimodal Imaging Assessment of Tissue Changes Following Poly‐L‐Lactic Acid Treatment for Midfacial Rejuvenation: An Exploratory Pilot Study
Source: J Cosmet Dermatol. 2026 Sep 21;25(10):e71178. doi: 10.1111/jocd.71178 (PMC13594687; doi:10.1111/jocd.71178)
Supplement: Supplementary file 1 [file JOCD-25-e71178-s001.docx]

**Supplementary Table S1. Exploratory analyses of the relationship between administered PLLA amount and volumetric changes at different follow-up time points**

| **Follow-up Time Point** | **Hemiface-level Exploratory Correlation (r, n = 12)** | **P value** | **Subject-level Aggregated Exploratory Correlation (r, n = 6)** |
| --- | --- | --- | --- |
| Day 30 | 0.51 | 0.093 | 0.57 |
| Day 90 | 0.73 | 0.007 | 0.71 |
| Day 180 | 0.55 | 0.064 | 0.59 |
| Day 360 | 0.42 | 0.175 | 0.22 |

Footnote: Hemiface-level analysis was performed using individual hemifaces as the primary anatomical unit (n = 12). To address potential within-subject correlation between bilateral hemifaces, a subject-level sensitivity analysis was performed by aggregating bilateral administered PLLA amounts and volumetric changes within each participant (n = 6). Pearson correlation coefficients are presented. Given the limited sample size, subject-level results are reported descriptively.

The greatest difference between hemiface-level and subject-level analyses was observed at Day 360 (R = 0.42 vs. R = 0.22), which may reflect variability between participants and the inherent instability of correlation estimates in very small samples (n = 6).
